# Regional Trends for the 2021 COVID-19 Independent Plastic Surgery Match Cycle

**DOI:** 10.7759/cureus.29172

**Published:** 2022-09-14

**Authors:** Haris M Akhter, Lauren Weis, Cassie Huang, Kaeli K Samson, Philip McCarthy, Heidi Hon

**Affiliations:** 1 Plastic and Reconstructive Surgery, University of Nebraska Medical Center, Omaha, USA; 2 Biostatistics, University of Nebraska Medical Center, Omaha, USA

**Keywords:** regional trends, location, covid, plastic surgery, independent match

## Abstract

Introduction: The coronavirus disease (COVID) created an abrupt change to virtual experiences and interviews for both the integrated and independent plastic surgery match cycle of 2021. Studies have shown that during the 2021 match cycle, integrated applicants were more likely to match at their home institution and region of medical school. These geographic and location trends for the 2021 match cycle have not been explored yet for the independent plastic surgery match.

Methods: Information for independent plastic surgery applicants that successfully matched was gathered using publicly available data for the 2019 and 2020 pre-COVID and 2021 COVID match cycles. Zip codes for applicant medical school, applicant residency program, and plastic surgery program were gathered to compare regional and distance outcomes between the pre-COVID and COVID match cycles.

Results: Data was collected on 182 applicants from 42 programs. There was no significant difference in the breakdown of gender percentages between the COVID match cycle (63.2% males) and the pre-COVID match cycles (72% males) (p=0.23). The COVID match cycle had 38.6% of applicants match at a plastics program within the same region as their residency, while the pre-COVID match cycles had 47.2% of applicants match the same region (p=0.28). These results continued to be nonsignificant when stratified by the regions of the west, south, midwest, and northeast (p=1.00). With regional matches with respect to medical school, the COVID match cycle had 33.3% of applicants match at a plastics program within the same region as their medical school, while the pre-COVID match cycles had 43.6% (p=0.20). These results continued to be nonsignificant when stratified by the four regions (p=1.00). When comparing the median distances between the COVID match cycle and the pre-COVID match cycle, no region of the United States showed a significant difference in travel distance to a plastics program with respect to medical school or residency (p=1.00).

Conclusion: Transitions to virtual interviews and cancellation of away rotations during the COVID match cycle for the independent plastic surgery match did not significantly affect an applicant’s ability to match outside of their region of previous medical school or residency. This may represent diminished program preference for applicants within the same region as their plastic residency.

## Introduction

There are two main avenues to becoming a plastic and reconstructive surgeon. The first and now more common pathway is the integrated model. This model entails 6-7 years of training after medical school, and it is known to be one of the most competitive residencies to match, with a match rate of 56% in 2021 [1]. The second option is the independent model. This model entails three years of additional training after completing any of the following surgical residencies: general surgery, orthopedic surgery, otolaryngology, neurosurgery, urology, and oral and maxillofacial surgery (with two years of general surgery). The overwhelming majority of applicants for the independent model are general surgery trained; the match rate in 2021 was also competitive at 70% [2].

The coronavirus disease (COVID) pandemic from 2019 to the present day presented a challenge for applicants in the 2020-2021 match cycle for both training models. Firstly, integrated applicants were unable to perform in-person away rotations. Secondly, applicants had all their interviews performed virtually. About half the applicants in the independent model were able to complete in-person away rotations and in-person interviews, but COVID restrictions created an abrupt change in the middle of the cycle with a transition to virtual experiences. Studies have continued to demonstrate that for the integrated model, applicants within the COVID match cycle were more likely to match at their home institution compared to previous match cycles [3-8]. Additionally, preexisting program connections and regional preferences played a more significant role for these integrated applicants [3,7].

There has not been published literature thus far comparing COVID match cycle outcomes for independent applicants. Prior to COVID, over the last decade, the number of available positions in the independent model has declined with program preference in converting independent programs to the integrated model [2,9]. The independent training model is crucial for applicants who develop an interest in plastic surgery during residency, and current research shows that interest in independent training is not waning [10]. Therefore, it is important for independent applicants to be informed about the various application outcomes during the pandemic match cycle. Like the integrated match, we hypothesize that the independent applicants in the COVID match cycle will match into regional programs at higher rates than in previous pre-COVID match cycles.

## Materials and methods

Using publicly available plastic surgery program websites and social media, data for 2020-2021 (denoted 2021), 2019-2020 (denoted 2020), and 2018-2019 (denoted 2019) was obtained for independent applicants who successfully matched. Data included match year, zip code of plastic surgery program applicants matched at, zip code of residency, zip code of the medical school, and gender. The United States was divided into the following regions: west, midwest, south, and northeast. It was noted whether an applicant’s residency and medical school are in the same or different regions of the plastic surgery program they matched. The years 2019 and 2020 will be grouped as pre-COVID match cycles, and 2021 will be noted as the COVID match cycle.

Associations between categorical variables were assessed using chi-square tests or Fisher’s exact tests when the expected cell counts were low. Logistic regressions were run for each outcome of interest (plastics’ region matched residency region, plastics’ region match medical school region, and plastics’ region matched residency program) and included region, year of the match (dichotomized into pre-2021 or 2021), and the interaction between year and region. If the interaction between year and region was not significant, main effect models were run and interpreted. To be able to run a more granular analysis of distance, the geodetic distance (in miles) between the centroids of zip codes for medical, residency, and plastics programs were computed and summarized using medians and interquartile ranges (IQRs) for each time period and separately for each region. Differences between periods were assessed using Wilcoxon rank-sum tests for each region, and p-values were Bonferroni-adjusted to account for multiple comparisons. All analyses were performed using the SAS software version 9.4 (SAS Institute Inc., Cary, NC, USA).

## Results

Data was collected on 182 applicants from 42 programs. The exact year breakdown with gender demographic, same regional match for residency, and same regional match for medical school are displayed in Table 1. When dichotomizing the match years into COVID match cycle and pre-COVID match cycles, there was no significant difference in the breakdown of gender percentages between these two (COVID match cycle had 63.2% males and 36.8% females, and the pre-COVID match cycles had 72% males and 28% females) (p=0.23).

**Table 1:** Match Applicant Numbers by Year

Match Year	Gender		Matching Within the Same Region as:	
	Male	Female	Residency	Medical School
2019	41 (64.1%)	23 (35.9%)	34 (53.1%)	26 (40.63%)
2020	49 (80.3%)	12 (19.7%)	25 (50%)	28 (46.7%)
2021	36 (63.2%)	21 (36.8%)	22 (38.6%)	18 (33.3%)

When looking at the regional match with respect to residency, the COVID match cycle had 38.6% of applicants match at a plastics program within the same region as their residency, while the pre-COVID match cycles had 47.2% of applicants match the same region (p=0.28). When stratified by region in Table 2, there were no significant results indicating that a particular region of the United States had more applicants match within the same region in the COVID match cycle versus pre-COVID match cycles with respect to residency location. With regional matches with respect to medical school, the COVID match cycle had 33.3% of applicants match at a plastics program within the same region as their medical school, while the pre-COVID match cycles had 43.6% (p=0.20). When stratified by region in Table 3, there were no significant results indicating that a particular region of the United States had more applicants within the same region in the COVID versus pre-COVID match cycles with respect to medical school location.

**Table 2:** Regional Match From Residency P-values are derived from chi-square tests unless otherwise indicated. †Fisher’s exact test

Region Resident Matched Plastic Surgery		Region Resident Completed Prior to Residency		Bonferroni-Adjusted P-Value
		Same Region	Different Region	
West	COVID	1 (16.7%)	5 (83.3%)	1.00^†^
	Pre-COVID	2 (18.2%)	9 (81.8%)	
South	COVID Match	11 (47.8%)	12 (52.2%)	1.00
	Pre-COVID	30 (51.7%)	28 (48.3%)	
Midwest	COVID	3 (23.1%)	10 (76.9%)	1.00
	Pre-COVID	10 (40%)	15 (60%)	
Northeast	COVID	7 (46.7%)	8 (53.3%)	1.00
	Pre-COVID	17 (54.8%)	14 (45.2%)	

**Table 3:** Regional Match From Medical School P-values are derived from chi-square tests unless otherwise indicated. †Fisher’s exact test

Region Resident Matched Plastic Surgery		Region Resident Completed Prior to Residency		Bonferroni-Adjusted P-Value
		Same Region	Different Region	
West	COVID	0 (0%)	6 (100%)	1.00^†^
	Pre-COVID	1 (9.1%)	10 (90.9%)	
South	COVID Match	8 (36.4%)	14 (63.6%)	1.00
	Pre-COVID	25 (43.9%)	32 (56.1%)	
Midwest	COVID	3 (25%)	9 (75%)	1.00
	Pre-COVID	14 (56%)	11 (44%)	
Northeast	COVID	7 (50%)	7 (50%)	1.00
	Pre-COVID	14 (45.2%)	17 (54.8%)	

Distances (in miles) between an applicant’s residency and plastic surgery were calculated, stratified by region and match cycle, and displayed in Table 4. No region displayed a significant difference in median distance from residency between the COVID match cycle and pre-COVID match cycles. The results were displayed similarly in Table 5 for distance from medical school and stratified by region and match cycle; the results were not significant. There were no regions with a significant difference in median distance from the medical school region between the COVID and pre-COVID match cycles.

**Table 4:** Distance From Residency

Region Resident Matched Plastic Surgery		Median Distance From Residency (25^th^-72^nd^ Percentiles) (Miles)	Bonferroni-Adjusted P-Value From Wilcoxon Rank-Sum Test
West	COVID	1870.6 (824.9-2390.4)	1.00
	Pre-COVID	1613.7 (616.8-1899.6)	
South	COVID Match	633.6 (246.7-902.7)	1.00
	Pre-COVID	677.2 (227.1-1168.3)	
Midwest	COVID	487.7 (222.4-746.9)	1.00
	Pre-COVID	489.6 (239.4-757)	
Northeast	COVID	456.6 (8.7-1171.6)	1.00
	Pre-COVID	231.8 (84.9-737.2)	

**Table 5:** Distance From Medical School

Region Resident Matched Plastic Surgery		Median Distance From Medical School (25^th^-72^nd ^Percentiles) (Miles)	Bonferroni-Adjusted P-Value From Wilcoxon Rank-Sum Test
West	COVID	1635 (1464.5-2334.5)	1.00
	Pre-COVID	1895.2 (824.9-2176.6)	
South	COVID Match	586.1 (229.2-1051.5)	1.00
	Pre-COVID	680.5 (218.1-1048.7)	
Midwest	COVID	379.5 (130.3-1009.9)	1.00
	Pre-COVID	245.3 (118.5-528.3)	
Northeast	COVID	327 (111.4-1207.7)	1.00
	Pre-COVID	281.5 (90.7-794)	

## Discussion

Although not significant for the consecutive independent match cycles of 2019, 2020, and 2021, there has been a decreasing number of applicants matching within the same region as their residency. Before the COVID match cycle of 2021, there was a 12.1% drop in applicants matching within the same region as their residency from 2019 to 2020. This pre-COVID regional drop may represent the pressure applicants face due to declining independent positions each year, thus applying more broadly. While not the primary focus of this study, studying the application trends in the independent match may better inform future applicants to optimize their chances as positions continue to decrease.

In contrast to the integrated match, there was no significant difference during the COVID match cycle of applicants matching within the same region as their residency or medical school compared to pre-COVID match cycles. While not the expected result, similar outcomes have been seen in the urology match during the COVID match cycle, where there was no significant increase in applicants staying at or within the geography of their home program [11]. While not significant, the COVID match cycle did see a lower proportion of applicants go into the same plastics program as their residency or medical school than in non-COVID match cycles. Likewise, this trend continues when stratified by residency and medical school region, except for the northeast region regarding medical school. These results may be secondary to applicants applying more broadly due to declining positions and programs not being as selective for regional applicants. However, this may have been confounded because the COVID match cycle did not become virtual until part-way through; applicants who completed in-person rotations and interviews may be swaying the sample. Once data is available, the 2022 COVID match cycle should be analyzed independently, as this was all completed virtually.

Lastly, to reinforce the results of the regional outcomes, the distance of the plastics program was analyzed from an applicant’s medical school and residency region. While the results were not significant, the trend continues where COVID match cycle applicants tended to have higher median distances than non-COVID match cycles. This will be a valuable marker to follow as the pandemic wanes as this may be due to the decreasing number of positions each year, therefore requiring applicants to travel farther to their respective matched programs.

Limitations to this study design are reliance on up-to-date and available applicant match information from individual program websites. Some programs did not update their websites with current matched residents and did not have the complete demographic information listed. Some of this information could not be found publicly either. Additionally, the geographic regions of the completed residency and medical school and distances to a particular plastic surgery program can be viewed as surrogates for applicant connections. However, these do not give the complete picture. Applicants could have had preexisting relationships that factor into the match, whether through research or faculty connections. Combining the 2022 independent plastics match data into the COVID year can help paint a more apparent trend for future studies, especially considering that the virtual cycle was not fully implemented until the tail-end of the 2021 independent match. Additionally, continued survey data to independent plastic program directors can help provide prospective applicants about what is valued.

## Conclusions

Transitions to virtual interviews and cancellation of away rotations during the COVID match cycle for the independent plastic surgery match did not significantly affect an applicant’s ability to match outside of their region of previous medical school or residency. This may represent diminished program preference for applicants within the same region as their plastic residency. This may pose consideration for future applicants on balancing the cost of away rotations and applying broadly as they are now more likely to match outside of their region if desired.
